# Alteration of Behavioral Changes and Hippocampus Galanin Expression in Chronic Unpredictable Mild Stress-Induced Depression Rats and Effect of Electroacupuncture Treatment

**DOI:** 10.1155/2014/179796

**Published:** 2014-10-30

**Authors:** Yuping Mo, Haijiang Yao, Hongtao Song, Xin Wang, Wanshun Chen, Jiawula Abulizi, Anping Xu, Yinshan Tang, Xiangbo Han, Zhigang Li

**Affiliations:** ^1^School of Acupuncture, Moxibustion and Tuina, Beijing University of Chinese Medicine, No. 11 North Third Ring Road East, Chaoyang, Beijing 100029, China; ^2^Department of Traditional Chinese Medicine, Inner Mongolia People's Hospital, No. 20 Zhao Wuda Road, Hohhot, Inner Mongolia Autonomous Region 010017, China; ^3^Department of Traditional Chinese Medicine and Rehabilitation, The Second Affiliated Hospital of Zhejiang University School of Medicine, No. 88 Jiefang Road, Hangzhou, Zhejiang 310009, China

## Abstract

To explore new noninvasive treatment options for depression, this study investigated the effects of electric acupuncture (EA) for depression rat models. Depression in rats was induced by unpredictable chronic mild stress (UCMS) combined with isolation for 21 days. Eighteen male Sprague-Dawley rats were randomly assigned into three groups: control, model, and EA groups. Rats were treated by EA once daily for 21 days. The results showed that body weight and sucrose consumption were significantly increased in EA group than in the model group. The crossing numbers and rearing numbers in the open field test significantly decreased in the model group but not in the EA group. And EA treatments upregulated levels of hippocampus galanin (Gal) in UCMS rats back to relative normal levels. The present study suggested that EA had antidepressant effects on UCMS model rats. The potential antidepressant effect may be related to upregulating Gal expression in hippocampus.

## 1. Introduction

Depression is a kind of emotional disorder disease, characterized by low mood, body discomfort, and sleep disorders. Persistent state of depression, as in clinical expression, affects patients' daily life and social function. World Health Organization (WHO) forecasts that depression will be the 2nd highest disease to threaten human's health and increase the economic burden by 2020 [1].

The number of studies about acupuncture treatment for depression has increased in recent years. Electric acupuncture as a part of acupuncture treatment plays an important role gradually; effective treatments and further research are expected by many researchers and patients [2].

Neuropeptides are a kind of polypeptide which can transfer information. Hundreds of neuropeptides have been detected and found to extensively exist in nervous system and every part of the body. They take part in regulating many functions, like stress and cardiovascular activity. Many neuropeptide expressions are observed producing noticeable changes during the process of stress reaction, such as galanin (Gal), neuropeptide Y (NPY), vasopressin (VP), corticotrophin releasing hormone (CRH), substance p (SP), and somatostatin [3]. Neuropeptide is thought to be the upstream regulatory factor of monoamine neurotransmitter, which has great influence on neurotransmitter metabolism and function; its effect in the process of depression is the subject of much attention.

Galanin (Gal) is a kind of conservative neuropeptide. Research using ^125^I to mark Gal receptor showed that Gal and its binding sites were widely distributed in the whole central nervous system, and its immunocompetence showed to be relatively high in hippocampus, hypothalamus, and so on; another research found that Gal coexisted with acetylcholine, 5-HT, and NE such as classical neurotransmitter, and to adjust their release by hyperpolarization. The features mentioned indicate that Gal takes part in regulating many physiological functions and may be connected with cognition, emotional behavior, and other advanced physiological functions [4, 5].

The regulatory effect of Gal on neuroendocrine stress reactions and its involvement in anxious and depressive symptomatology have been thoroughly reviewed [6–10]. Animal behavioral studies [11–17] and a human study [18] suggest that Gal has a role in stress, depression like behavior, and anxiety. In addition, there is an indication from previous genetic studies on humans that the Gal system is involved in psychiatric disorders including alcoholism/addiction [19–23], panic disorder [6, 24], and chronic pain-associated depression [25]. Furthermore, recent functional studies provided the first evidence that polymorphisms in a highly conserved genetic region upstream from the Gal gene regulates Gal expression in brain areas, such as the amygdala and hypothalamus, implicated in the pathogenesis of depression [26, 27].

So, we think that Gal has a close relationship with depression, but there has been limited research on the effect of EA intervention to depression and its influence on Gal expression and its mechanism are not known at present.

Unpredictable chronic mild stress (UCMS) as a kind of effective depression model has been widely used in basic research and treatment selection of depression [28].

So this paper is aiming to use UCMS with isolation to establish depression model and use EA as intervention to treat depression. Detecting Gal mRNA and protein expression through real-time PCR and ELISA to explore the role of Gal in mechanism of rats' experimental depression and the effect of EA on them, the purpose is to provide new method and laboratory evidence for diagnosis and prevention of depression in clinic.

## 2. Material and Methods

### 2.1. Animals

A total of eighteen male Sprague-Dawley rats weighing 200 to 240 g were purchased from the Vital River Laboratory Animal Technology Co. Ltd. (Beijing, certificate no. SCXK(Jing)2012-0001). The animals were housed in cages at 22 ± 2°C and humidity of 45% ± 5% under a 12-hour light/dark cycle (lights on at 8:00 a.m., lights off at 8:00 p.m.) and received standard diet and water ad libitum. Animals were allowed to acclimatize for 3 days before the study. All experiment procedures comply with the guidelines of the “Principles of Laboratory Animal Care” formulates by the National Institute of Health and the legislation of China for the use and care of laboratory animals. The experimental protocols were approved by the Animal Experimentation Ethics Committee of Beijing University of Chinese Medicine. Efforts were made to minimize the number of animal use and the suffering of the experimental animals.

### 2.2. Groups and Unpredictable Chronic Mild Stress (UCMS) Model

Eighteen rats were distributed into three groups: the control group (*n* = 6), no model induction and treatment were performed; the model group (*n* = 6), UCMS were conducted for 21 days without treatment; the EA group (*n* = 6), EA pretreatment was conducted daily prior to UCMS for 21 days. Every six rats in the control group were housed in one cage. However, rats in the model and EA groups were caged individually. Depression model was established by 21 days of UCMS combined with isolation. UCMS procedures contain food deprivation (24 h), water deprivation (24 h), wet bedding (24 h), swimming in 4°C ice water (5 min), clipping tail (5 min), 100 V electric shock (2 mA, 5 min), reversed light/dark cycle (12 h), randomly assigned 1 kind of stimulation daily, and each stimulus repeated 3 times.

### 2.3. EA Treatment

**Figure 1:**
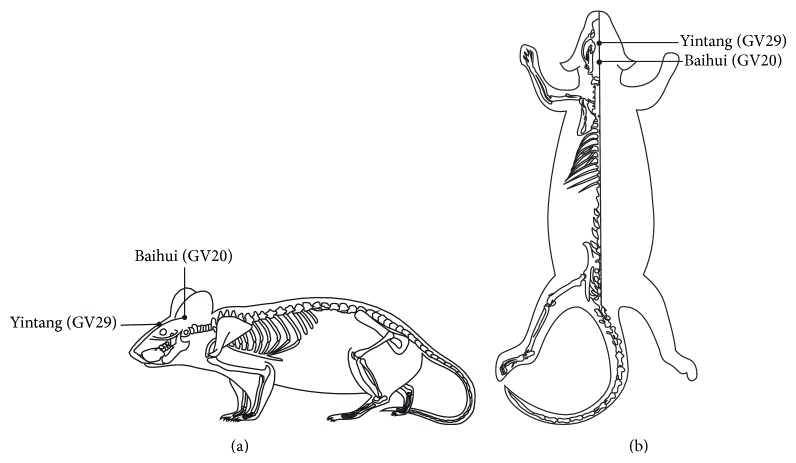
Drawings (a) and (b) show where electroacupuncture was applied.

During acupuncture administration, rats were maintained within a cloth bag. Two points were selected: Baihui (GV20) and Yintang (GV29). GV20 is located above the apex auriculate, on the midline of the head. GV29 is located at the middle point between two eyes [29] (Figure 1). Sterilized disposable stainless steel needles (0.30∗25 mm, Hua Tuo brand, manufactured by Tianjin Xinlinshuyuan Medical Devices Co., Ltd., Tianjin, China) were inserted obliquely as deep as 0.5–1 cm for both points. Following the insertions, electrodes were added to the handle of the needles (electric acupuncture apparatus used: LH202acupoint nerve stimulator, manufactured by Beijing Huawei Industrial Development Co., Ltd., Beijing, China). Electricity simulation parameters were 1 mA, 2 Hz, for 20 minutes.

### 2.4. Open Field Test, Sucrose Consumption, and Body Weight

The open field apparatus was constructed of black wood and measured 100∗100 cm with 50 cm walls. White lines were drawn on the floor. The lines divided the floor. The lines divided the floor into twenty-five 20∗20 cm equal squares. A single rat was gently placed in the center square to explore the arena for 5 min, at the same time the video camera was turned on for video recording from the top of the open field apparatus. Two observers, blind to the experiment, counted the crossing numbers (defined as four paws in a square) and the rearing numbers (defined as both frontal claws uplifting from the group). The open field test was conducted at day 0 and day 22. The time of each test was fixed at 9 a.m. to 11 a.m. and was performed in a room which was with sound and light insulation and constant temperature.

The sucrose consumption test was conducted at day 0, day 7, day 14, and day 21. This method was adopted elsewhere with slight modifications [30]. The rats had free access to sucrose solution (1% w/v) for 3 days from the commencement of dosing for habituation. Before the test, water deprivation for 24 h to the rats, they were given 1% sucrose solution, totally 250 g with bottle. Then the rats were given a 24 h window sucrose test. The sucrose consumption was measured by reweighing preweighed bottles of sucrose solution. Stressors were not applied during the period of sucrose intake measurement. Control rats were housed individually during the test.

The body weight was measured on day 0, day 7, day 14, and day 21 of the experiment.

### 2.5. Real-Time Polymerase Chain Reaction Detection of Gal mRNA Expression

Rats were sacrificed at the 22nd day of the experiment for the detection of GAL mRNA expression, using the real-time polymerase chain reaction method. Rat was quickly decollated and the brain was collected on the ice plate; then hippocampal tissue was isolated and placed into 1.5 mL tube and stored in liquid nitrogen temporarily and then transferred to −80°C refrigerator. Total RNA extraction from the specimens was performed according to the instructions of the Trizol Reagent. Reverse transcription was conducted according to the instruction of Promege Reverse Transcription System. Primers were designed and synthesized by Sangon Biotech (Shanghai) Co., Ltd., (GAL: 5′-CCTGAGACCACACCCACTGT-3′ and 5′-CAGCATCAAAGCAGAGAACAAA-3′; and *β*-actin: 5′-ACCGTGAAAAGATGACCCAGAT-3′ and 5′-CCAGAGGCATACAGGGACAA-3′, forward and reverse, resp.). The polymerase chain reaction was performed according to the instructions of the Applied Biosystems Step One Plus Real-Time PCR System version 2.1. In brief, the cycle conditions were 95°C for 10 min and 95°C for 5 sec, followed by 40 cycles at 60°C for 30 sec, 95°C for 15 sec, 60°C for 1 min, and at 95°C for 15 sec. Fold-changes in gene expression were estimated using the CT comparative method normalizing to *β*-actin CT values and relative to control samples as follows: ΔCt = Ct GAL (NPY)-Ct *β*-actin; ΔΔCt = ΔCt − ΔCt control; fold difference = 2^−(ΔΔCT)^.

### 2.6. Enzyme Linked Immunosorbent Assay Detection of Gal Protein Expression

Rats were sacrificed at the 22nd day of the experiment for the detection of GAL protein expression, using the enzyme linked immunosorbent assay (ELISA) method. Rat was quickly decollated and the brain was collected on the ice plate, then hippocampal tissue was isolated and placed into 1.5 mL tube and stored in liquid nitrogen temporarily and then transferred to −80°C refrigerator. Hippocampal was weighed and added 1XPBS (Phosphate Buffer Solution) 400 *μ*L to homogenate and then added 400 *μ*L PBS again after homogenate, placed on the ice 20 min (oscillation 5 s every 5 min), centrifuged at 10000 ×g for 5 min at 4°C, and repackaged protein, and concentration of protein was detected according to the instructions of Bicinchoninic acid (BCA) (Applygen, Beijing, China). The Gal protein in rats' hippocampal was detected according to the operating steps of CUSABIO Rat galanin (GAL) ELISA Kit (Huamei, Wuhan, China) and dividing the concentration of Gal, NPY by the total concentration of protein to statistical analysis.

### 2.7. Statistical Analysis

Data were presented as means ± SEM. SPSS 20.0 (SPSS Inc, Chicago, USA) was deployed for data analysis with one-way ANOVA method after the test of normal distribution and homogeneity of variance, followed by post hoc multiple comparison. Between the two groups, we used the LSD method to compare any difference. Statistical significance was set to *P* < 0.05, while highly statistical significance was set to *P* < 0.01.

## 3. Results

### 3.1. Effects of EA Treatment on Body Weight

**Figure 2:**
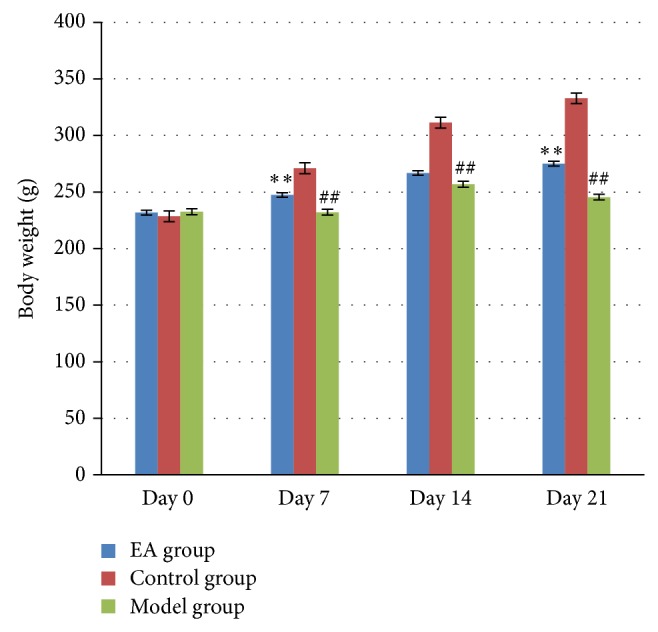
The effect of electric acupuncture (EA) on body weight at selected time points in the following groups (*n* = 6 per group): EA, control, and model. ^##^ *P* < 0.01 as compared with control group; ^**^ *P* < 0.01 as compared with model group.

As shown in Figure 2, the body weight increased slowly in model group and EA group in contrast with that in control group. Twenty-one days after induction, the body weights in model group were significantly lower compared with that in control group (*P* < 0.01), whereas the body weights in EA group were significantly increased in day 7 and day 21 in comparison with that in model group (*P* < 0.01). This result indicated that UCMS has a negative influence on body weight, while EA can reverse this change which means EA has a positive effect on body weight.

### 3.2. Effects of EA Treatment on Sucrose Consumption

**Figure 3:**
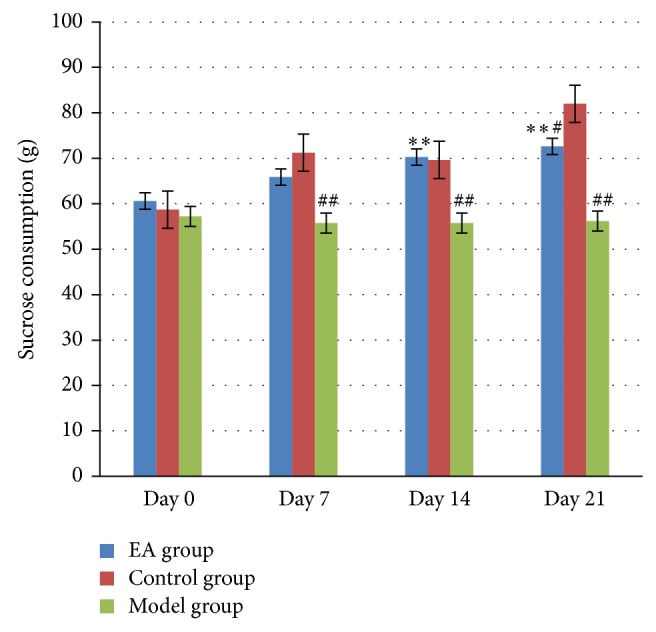
The effect of electric acupuncture (EA) on sucrose consumption at selected time points in the following groups (*n* = 6 per group): EA, control, and model. ^##^ *P* < 0.01 as compared with control group; ^#^ *P* < 0.05 as compared with control group; ^**^ *P* < 0.01 as compared with model group.

The sucrose consumption test is often used to measure depression-like behavior in rats by the evaluation of the hedonic state or the ability to gain pleasure [31]. As shown in Figure 3, the sucrose consumption in control group displayed an increased tendency, while those in model group and EA group increased slowly, and sucrose consumption in model group was less than that in control group with statistically significant differences (*P* < 0.01) at day 7, day 14, and day 21, whereas the sucrose consumption in EA group was much more than that in model group with statistically significant differences (*P* < 0.01) at day 14 and day 21 but still less than that in control group with statistical differences (*P* < 0.05) at day 21. This result suggested that rats lost interest in sucrose which also called reward after 21 days UCMS induction, and EA can improve this situation.

### 3.3. Effects of EA Treatment on Open Field Test

**Figure 4:**
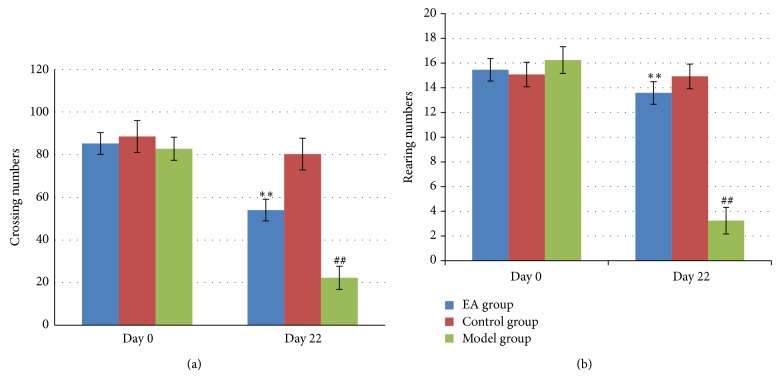
The effect of electric acupuncture (EA) on crossing numbers and rearing numbers in open field test at day 0 and day 22 in the following groups (*n* = 6 per group): EA, control, and model. (a) Crossing numbers in open field test. (b) Rearing numbers in open field test. ^##^ *P* < 0.01 as compared with control group; ^**^ *P* < 0.01 as compared with model group.

Locomotors activity and exploratory behavior are evaluated using an open field test [32, 33]. As shown in Figures 4(a) and 4(b), 21 days after induction of the crossing numbers and rearing numbers, model group was significantly decreased in comparison with those in control group with statistically significant differences (*P* < 0.01). It indicated that UCMS model was established, while the crossing numbers and rearing numbers in EA group were increased remarkably in comparison with those in model group with statistical significance (*P* < 0.01). This result suggested that EA plays an important role in ameliorating stress-impaired exploratory and locomotor activities.

### 3.4. Effects of EA Treatment on GAL mRNA

**Figure 5:**
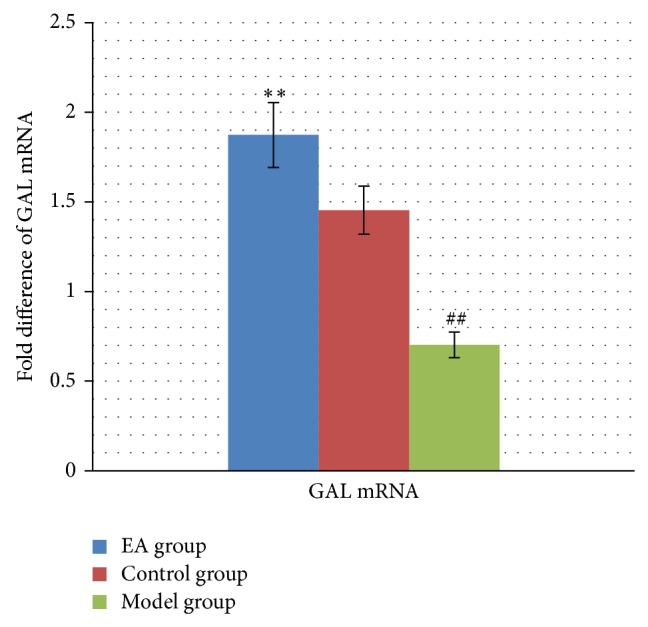
The effect of EA on GAL mRNA expression in hippocampus in the following groups (*n* = 6 per group): EA, control, and model. ^##^ *P* < 0.01, as compared with control group; ^**^ *P* < 0.01, as compared with model group.

As shown in Figure 5, GAL mRNA expression in hippocampus of model group decreased significantly in comparison with that in control group with statistically significant differences (*P* < 0.01), while GAL mRNA expression in hippocampus of EA group was increased remarkably in comparison with those in model group with statistical significance (*P* < 0.01). These results indicated that EA increased the expression of GAL in hippocampal tissue.

### 3.5. Effects of EA Treatment on GAL Protein Expression

**Figure 6:**
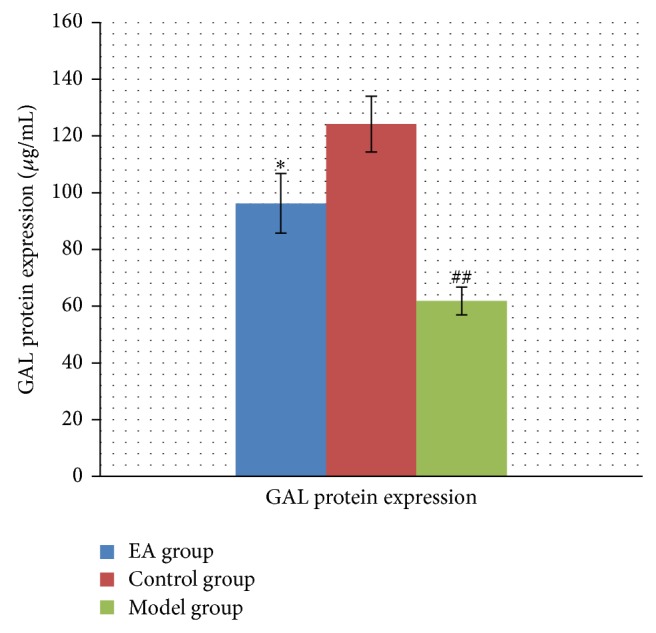
The effect of EA on GAL protein expression in hippocampus in the following groups (*n* = 6 per group): EA, control, and model. ^##^ *P* < 0.01, as compared with control group; ^*^ *P* < 0.05, as compared with model group.

As shown in Figure 6, GAL protein expression in hippocampus of model group decreased significantly in comparison with that in control group with statistically significant differences (*P* < 0.01), while GAL protein expression in hippocampus of EA group was increased in comparison with those in model group with statistical significance (*P* < 0.05). These results indicated that EA increased the expression of GAL in hippocampal tissue.

## 4. Discussions

The present study aims to use UCMS with isolation to establish depression model. This model, which has features of stress factor changeable and unpredictable, is closely related to the mechanism of occurrence and development of depression in human. After 21 days of induction, in model group, rats' body weight increased slowly, rewarding action weakened, and locomotor activities and explore interests reduced; while EA group could reverse this change, it indicated that the established depression model was reliable, and EA had positive effect to depression; we could come to the next step to analyze the mechanism.

The hippocampus is closely related to learning, memorizing, behavior, and emotion. It is also an important target of stress-induced injury [34].

The Gal had been studied for its role in stress and depression. Many animal experimental research showed that increasing Gal signal transduction had the effect of antidepression [35, 36]. For example, preclinical studies had suggested that depression was associated with aberrant Gal expression, chronic stress exposure caused the reduction in Gal in the hippocampus of rat [37, 38], and giving antidepressive therapy could increase them [37, 38]. Holmes [39] has also shown that exercise exerts antidepressant effects in chronic models of depression [40], and chronic antidepressant treatment elevates Gal mRNA in the locus coeruleus (LC) similarly to exercise [41]. On the other hand, controversial studies exist. For example, it had been reported that Flinders sensitive line depression model induced an increase in Gal expression in dorsal raphe nucleus (DRN) [42] and UCMS induced an increase in Gal gene expression in amygdale and LC [42]. Shan [43] and Gao's [44] research showed that the content of Gal in experimental depression rats' hippocampus increased, but giving Gal and its receptor agonist could enhance rats' ability of independent activities and raise learning capacity. The possible explanation for the discrepancy may lie in the difference in stress category, duration, and other experimental procedures [45].

In consistency with most of these previous findings, we found that CUMS decreased Gal levels in the hippocampus. EA treatment could improve rats' behavior of depression model and increase the expression of Gal; it indicated that the increase of Gal content was a kind of protective regulation to depression [43, 44]. The results were similar to that of Lu et al.'s [46] research. Previous studies had demonstrated that antidepressant treatments strongly elevated Gal mRNA levels, which might be associated with induction of Gal synthesis and release [46]. Gal has three kinds of subtype receptors: GalR1–3, which has varying degrees of expression in central and peripheral tissue. GalR1 mRNA expressed in all of central area, expression in amygdale, and hypothalamus especially high [47]; GalR2 mRNA expressed relatively high in hippocampus, hypothalamus, cortex, and amygdale; GalR3 mRNA expressed high in hippocampus and hypothalamus, while expressed comparatively low in cortex and amygdale [48]. Concurrent with the increase in Gal peptide expression, an increase in GalR2-binding sites after antidepressant treatment was also found [46]. Indeed, excitatory effects of Gal, exerted probably through GalR2, on neurotransmitter release had been reported in some brain regions [46]. The use of Gal receptor antagonist, M40, underscored the findings that the increased Gal mRNA and GalR2 were relevant for the antidepressant-like effect [46]. The Gal receptor agonist, galnon, a nonselective agonist for GalR1/GalR2, was also used to underscore its acute antidepressant-like effect, which was probably similar to the effects of increased Gal release, enhanced by antidepressant treatment [46].

Subgranular zone (SGZ) of dentate gyrus in hippocampus is the place where adult mammalian's neural stem cell (NSC) regenerate; we can see high expression of Gal in this region. Gal combines with GalR2 which is on the membrane of NSC of SGZ, to activate PKC-ERK/MAPK signal pathway, induce NSC to mitosis, proliferation, and directional differentiation, then promote new neurons of SGZ brain region to survival, regeneration, and maturity and transfer to dentate gyrus, and then finish this neural circuitry integration [49–51]. Furthermore, activation of dorsal hippocampal GalR2 receptors facilitates cognition whereas activation of GalR1 in the ventral hippocampus impairs cognitive performance [52, 53], suggesting that a change in the overall action of Gal would occur when the balance between GalR1 and GalR2 is altered [46]. Therefore, we speculated that the reason which EA treatment could increase the expression of Gal might be that EA could activate GalR2 in the dorsal area of hippocampus to protect and promote the formation of cognitive function then to antidepression [38, 54]. The combined data suggested that the galaninergic system was a putative target for EA treatment, and further researches on various animal models and clinical studies by using selective Gal receptor ligands would be needed to validate our conclusion.

According to Traditional Chinese Medicine, Baihui and Yintang are points pertaining to Governor Meridian. Brain and Governor Meridian have a direct contact on channels and collaterals. Based on the principle of Meridians pass, indications considered selecting Governor Meridian to treat mind illnesses related to brain, conforming to the Traditional Chinese Medicine saying “searching for the primary cause of disease in treatment.” Meanwhile, EA treatment design of our study embodies one of the most critical theories in Traditional Chinese Medicine, the principle of “treating diseases prior to its onset” which attaches great significance on disease preventions [55].

## 5. Conclusion

In summary, the present study demonstrated that Gal in rats' hippocampus mediated the onsets of depressive symptoms after UCMS inductions. Importantly, our findings indicated that EA can significantly mitigate deficit behavioral activities elicited by UCMS through a potential mechanism of neuropeptide (Gal) modulation.

## Limitation

This pilot EA study on depression has a small sample size. And the interaction among neuropeptides (Gal) and their receptors (such as GalR1–3) in different parts of brain and the effect of EA to them should be explored in the future.
